# Mapping the global research landscape on molecular mimicry: a visualization and bibliometric study

**DOI:** 10.1186/s12967-024-05357-7

**Published:** 2024-06-03

**Authors:** Sa’ed H. Zyoud

**Affiliations:** 1 Department of Clinical and Community Pharmacy, College of Medicine and Health Sciences, An-Najah National University, Nablus, 44839 Palestine; 2 Clinical Research Centre, An-Najah National University Hospital, Nablus, 44839 Palestine

To the Editor,

This letter represents my interest in participating in the recently released Journal of Translational Medicine Collection on Molecular Mimicry in Human diseases. A better understanding of many human diseases is possible, and new therapeutic approaches based on molecular mimicry have been developed. Damian first described “molecular mimicry” in 1964. This theory suggests that pathogenic microbes may escape the immune system by expressing antigens that are closely related to those found in human hosts [1]. Since then, growing data from experimental and epidemiological studies have supported the connection between autoimmune and infectious diseases. This suggests that molecular mimicry and the resulting cross-reactivity are very important [2]. A better understanding of molecular mimicry in this environment will greatly influence the diagnosis, prevention, and treatment of these diseases [3].

Numerous disciplines have employed bibliometric analysis to identify and emphasize the most significant countries, institutions, journals, and citations [4, 5]. The application of mathematical and statistical techniques to quantitatively analyze and characterize published literature, provide evidence to support the formation of future research hotspots, master the discipline’s development trend, track the frontier of scientific research, improve the efficiency of scientific research, and propose research directions is known as bibliometrics [6]. It also summarizes the state of affairs and highlights hotspots in particular research fields. On the other hand, no bibliometric analysis of molecular mimicry has been performed. Therefore, the objective of this study was to conduct an in-depth review of the scientific advances in molecular mimicry. Consequently, the purpose of this bibliometric analysis was to investigate molecular mimicry research trends and to pinpoint potential future research hotspots. Furthermore, by offering references and concepts for further research on molecular mimicry pathogenicity, autoimmunity, and clinical applications, this study contributes significantly to the body of knowledge.

The current study uses the Scopus database, which is widely regarded among researchers for the purposes of high-quality bibliometric studies [7], although a significant number of databases are used for evaluative research at the global level [8]. The largest abstract and citation database of peer-reviewed research literature in the world, Scopus, is a trusted source for locating biomedical research, including MEDLINE documents. Because I am more interested in molecular mimicry as a novel idea in study than in related subjects, I used the terms “molecular mimicry” and its synonyms as my key words. The MeSH terms in PubMed and the findings of earlier studies on molecular mimicry were used to determine the relevant keywords [3, 9–11]. The date of data mining was May 14, 2024. The main focus was on journal articles that used “molecular mimicry” to identify objects based on searches conducted in the fields of titles and abstracts.

To understand research trends, a quantitative analysis of the obtained articles was carried out. A comprehensive picture of global research production was obtained by analyzing important indicators such as publication rates, the distribution of articles in journals, and institutional and geographical origins. The VOSviewer software (version 1.6.20) was used to create visual maps based on the most common terms in the title of the article and the abstract, further clarifying research areas and emerging themes [12]. This visualization approach offers important new perspectives on current hot topics of research and suggests possible paths for molecular mimicry.

Our search strategy identified 3,391 articles on molecular mimicry published between 1965 and 2023. Most of the research articles were of original type (63.78%, *n* = 2117), followed by reviews (30.94%, *n* = 1027) and other types (7.44%, *n* = 247), such as letters and editorials. As shown in Fig. 1, there was a strong positive correlation (*r* = 0.876, *p* < 0.001) between the publication year and the number of articles on molecular mimicry. Initially (1965–1990), there were few publications (annual average of approximately 7). However, from 1991 onward, there was a significant increase in publications (annual average of approximately 97), reaching a peak of 182 articles in 2023.

**Fig. 1:**
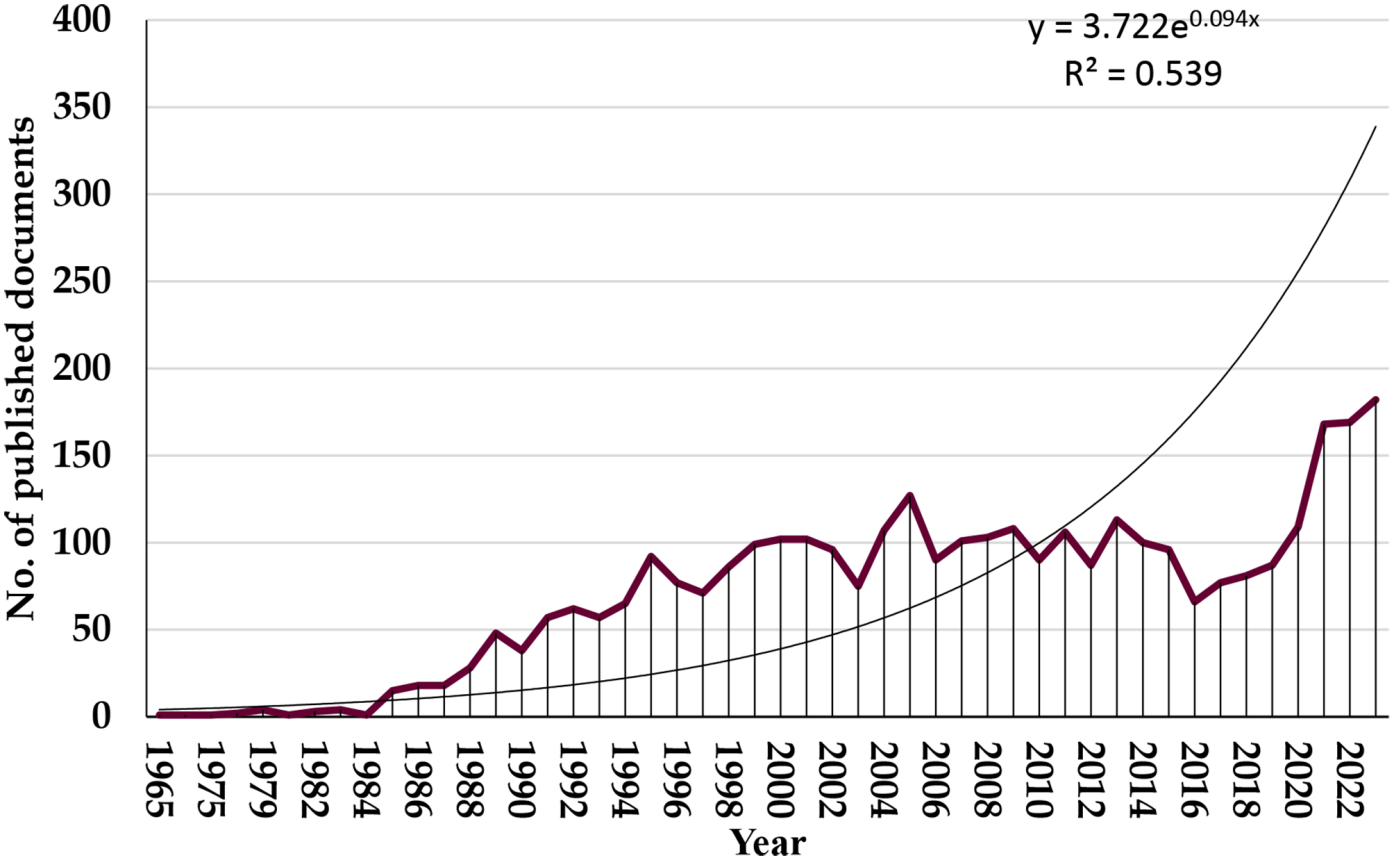
Annual number of publications related to molecular mimicry from 1965 to 2023

A worldwide review of publications on molecular mimicry research indicates a concentration in the US. With 1186 articles (34.97%) among the 94 contributing nations, the USA took the lead, followed by the UK (316, 9.32%), Italy (310, 9.14%), and other countries (Table 1). The *National Institutes of Health* (NIH) published 73 articles (2.15%), followed by *Tel Aviv University* (*n* = 56; 1.65%). American institutions dominate the field. *King’s College London* is another notable contributor (*n* = 53; 1.56%). The *Journal of Autoimmunity* (70 articles, 2.06%), *Journal of Immunology* (70 articles, 2.06%), *Frontiers in Immunology* (52 articles, 1.53%), and *Autoimmunity Reviews* (50 articles, 1.47%) are the most active journals that publish research on molecular mimicry.

**Table 1:** List of the top ten countries publishing research on molecular mimicry from 1965 to 2023

Ranking	Country	Number of documents	%
1st	United States	1186	34.97
2nd	United Kingdom	316	9.32
3rd	Italy	310	9.14
4th	Germany	235	6.93
5th	Japan	226	6.66
6th	China	168	4.95
7th	France	159	4.69
8th	Canada	151	4.45
9th	Australia	133	3.92
10th	India	125	3.69

The most cited article by Wucherpfennig and Strominger [13], published in *Cell* in 1995, investigated the role of molecular mimicry in T-cell-mediated autoimmunity. This study tested 129 peptides on seven myelin basic protein-specific T-cell clones from multiple sclerosis patients. Although only one peptide was identified as a molecular mimic, seven viral peptides and one bacterial peptide activated three of the clones. This finding suggested that a single T-cell receptor can recognize distinct but structurally related peptides from multiple pathogens, potentially contributing to the development of autoimmunity.

Three main areas of molecular mimicry research were identified by a thematic analysis of the literature (Fig. 2A):

**Fig. 2:**
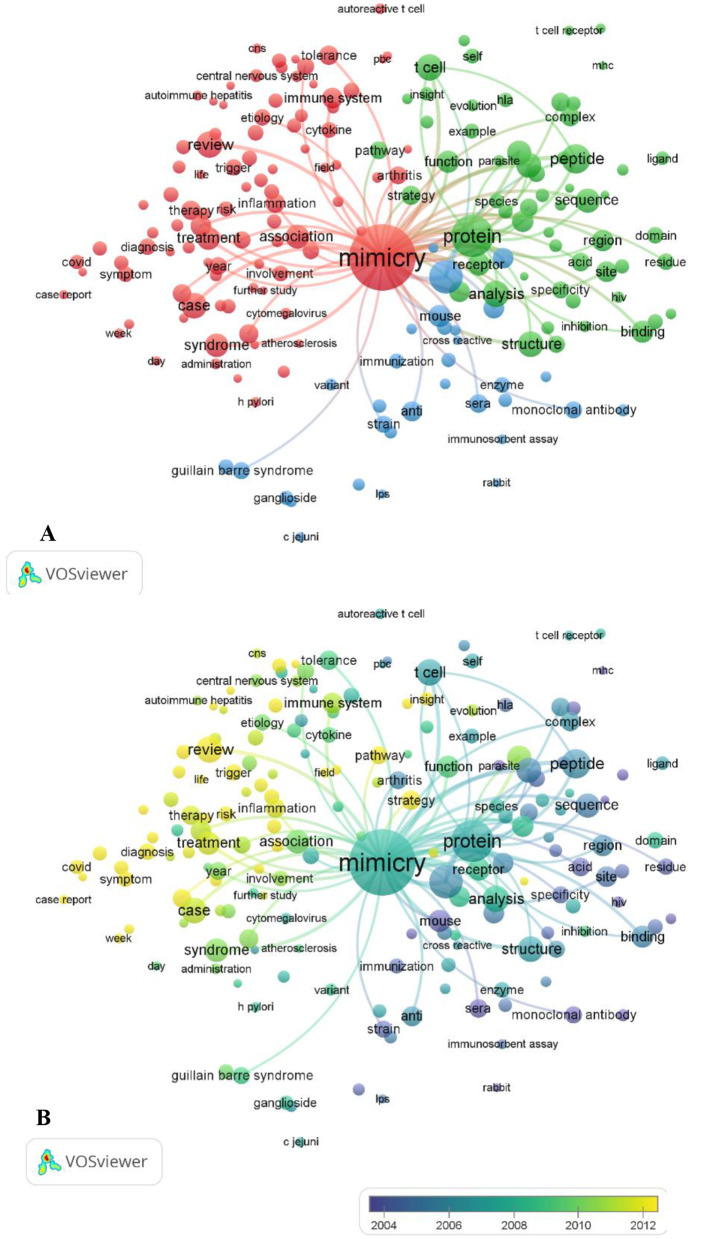
Mapping of terms used in research on molecular mimicry. **A**: Research topics clustered by mapping the cooccurrences of terms for publications on molecular mimicry. Of the 59,752 terms, 305 terms occurred at least 50 times. **B**: Overlay visualization map of the time sequence of frequently used terms in molecular mimicry (1965–2023). The yellow terms represent the most recent research

## Molecular mimicry function in autoimmune diseases (blue cluster)

This group investigates how the immune system can become confused by pathogen molecules that have structures similar to those of our own cells. Rheumatoid arthritis and Guillain–Barré syndrome are examples of autoimmune diseases that can arise when the immune system targets these ‘mimicked’ self-cells. The purpose of this research was to discover possible targets for treatment and elucidate the role that molecular mimicry plays in autoimmunity.

## Molecular mimicry and vaccine development (green cluster)

This group looks into the possibility of creating vaccines through molecular mimicry. Vaccines have the potential to induce a potent immune response by mimicking particular pathogen molecules without actually triggering the disease. These studies were aimed to develop safe and efficient mimicry-based vaccines as well as optimal mimicry targets.

## Targeting, mimicry discovery through specificity and computational tools (red cluster)

This group addresses the difficulty of attaining accurate targeting in molecular mimicry research. Important aspects include creating computational tools to detect highly specific mimics and ensuring that these mimics do not cause unwanted immune responses. Here, research has attempted to improve targeting tactics and make use of computing power to speed up the detection of mimics. Researchers are looking for ways to improve the specificity and design therapies that target specific mimics involved in the disease.

To illustrate the distribution of keyword frequencies across publications, I classified keywords by publication year and assigned them the corresponding colors, as shown in Fig. 2B. The blue keywords represent research areas emphasized in earlier studies (before 2012), while the yellow keywords highlight topics addressed in more recent publications (post 2012). Notably, keywords related to “targeting, mimicry discovery through specificity, and computational tools” emerged as a prominent theme after 2012, suggesting their growing importance for future research. On the contrary, research prior to 2012 appears to have focused more on “molecular mimicry and vaccine development” and “molecular mimicry’s function in autoimmune diseases”.

In conclusion, molecular mimicry has enormous potential for improving our understanding and ability to treat a wide range of human diseases. There has been a significant increase in publications on molecular mimicry since 1991, indicating a growing interest and activity in this area of research. After 2012, keyword analysis revealed a shift in research focus toward specificity and computational tools, indicating a growing emphasis on improving therapeutic and targeting approaches. In addition to their academic value, the findings of this study emphasize the importance of molecular mimicry in understanding disease mechanisms and developing therapeutic interventions, with implications for future research directions and practical applications.

## Data Availability

All the data generated or analyzed during this study are included in this published article. In addition, other datasets used during the current study are available from the author upon reasonable request (saedzyoud@yahoo.com).
